# Monoamine oxidase-A activity is required for clonal tumorsphere formation by human breast tumor cells

**DOI:** 10.1186/s11658-019-0183-8

**Published:** 2019-11-12

**Authors:** William D. Gwynne, Mirza S. Shakeel, Jianhan Wu, Robin M. Hallett, Adele Girgis-Gabardo, Anna Dvorkin-Gheva, John A. Hassell

**Affiliations:** Department of Biochemistry and Biomedical Sciences, McMaster University, Hamilton, Ontario Canada

**Keywords:** Breast tumor-initiating cells, Monoamine oxidase-A, Tumorspheres

## Abstract

**Background:**

Breast tumor growth and recurrence are driven by an infrequent population of breast tumor-initiating cells (BTIC). We and others have reported that the frequency of BTIC is orders of magnitude higher when breast tumor cells are propagated in vitro as clonal spheres, termed tumorspheres, by comparison to adherent cells. We exploited the latter to screen > 35,000 small molecules to identify agents capable of targeting BTIC. We unexpectedly discovered that selective antagonists of serotonin signaling were among the hit compounds. To better understand the relationship between serotonin and BTIC we expanded our analysis to include monoamine oxidase-A (MAO-A), an enzyme that metabolizes serotonin.

**Methods:**

We used the Nanostring technology and Western blotting to determine whether MAO-A is expressed in human breast tumor cell lines cultured as tumorspheres by comparison to those grown as adherent cells. We then determined whether MAO-A activity is required for tumorsphere formation, a surrogate in vitro assay for BTIC, by assessing whether selective MAO-A inhibitors affect the frequency of tumorsphere-forming cells. To learn whether MAO-A expression in breast tumor cells is associated with other reported properties of BTIC such as anticancer drug resistance or breast tumor recurrence, we performed differential gene expression analyses using publicly available transcriptomic datasets.

**Results:**

Tumorspheres derived from human breast tumor cell lines representative of every breast cancer clinical subtype displayed increased expression of MAO-A transcripts and protein by comparison to adherent cells. Surprisingly, inhibition of MAO-A activity with selective inhibitors reduced the frequency of tumorsphere-forming cells. We also found that increased MAO-A expression is a common feature of human breast tumor cell lines that have acquired anticancer drug resistance and is associated with poor recurrence-free survival (RFS) in patients that experienced high-grade, ER-negative (ER^−^) breast tumors.

**Conclusions:**

Our data suggests that MAO-A activity is required for tumorsphere formation and that its expression in breast tumor cells is associated with BTIC-related properties. The discovery that a selective MAO-A inhibitor targets tumorsphere-forming cells with potencies in the nanomolar range provides the first evidence of this agent’s anticancer property. These data warrant further investigation of the link between MAO-A and BTIC.

## Background

Recent studies demonstrate that breast tumors comprise an infrequent stem-like tumor cell population, termed BTIC or breast cancer stem cells, which initiate and sustain tumor growth, seed metastases and resist cytotoxic therapies [1–3]. Whereas identifying agents capable of eradicating these cells would significantly improve breast cancer (BC) survival, achieving the latter has been challenging due largely to their scarcity in primary tumors [4].

We previously reported BTIC frequencies ranging between 20 and 50% in tumors arising in 3 different transgenic mouse models of BC [5]. Propagation of the primary mammary tumor cells in vitro in chemically defined, serum-free media as non-adherent tumorspheres preserves the high BTIC fraction found in the primary tumors, whereas culturing the tumor cells in serum-containing media as adherent cells reduced BTIC frequencies by 4–5 orders of magnitude [5]. Others have also shown that culturing cells from human breast tumors and breast tumor cell lines as tumorspheres similarly increases BTIC frequencies [6, 7].

The high BTIC frequencies in mouse mammary tumorspheres encouraged us to perform a high-throughput phenotypic screen to identify small molecules that inhibit their activity [8]. One class of compounds identified in the screen are antagonists of neurotransmitter activity, including selective antagonists of serotonin receptors and the serotonin reuptake transporter (SERT). We subsequently established a connection between serotonin and BTIC by demonstrating that mouse [8] and human [9] breast tumor cells synthesize serotonin and that antagonists of SERT inhibit BTIC activity using multiple orthogonal assays and synergize with chemotherapy to inhibit the growth of breast tumor allografts and xenografts in vivo.

To better understand the link between serotonin and BTIC we expanded our analyses to include other serotonin pathway proteins that were not identified in our screen and which we had not previously investigated, namely MAO-A, a mitochondrial enzyme that metabolizes serotonin [10] and whose expression and activity are required for prostate TIC activity [11, 12]. To this end we cultured human breast tumor cell lines modeling each of the BC clinical subtypes in chemically defined media as tumorspheres and in serum-containing media as adherent cells. We found that MAO-A transcripts and protein were more highly expressed in tumorspheres by comparison to adherent cells. Moreover, we found that treatment of tumorsphere-derived cells with selective MAO-A inhibitors reduced the frequency of tumorsphere-forming cells implying that its activity is required for this process.

We suspected that increased MAO-A expression might be associated with other properties of BTIC such as acquired anticancer drug resistance [2] or the tumors of patients who experienced a poor prognosis [3]. To explore the latter, we performed differential gene expression analyses using publicly available datasets and found that increased MAO-A transcript expression is a feature of breast tumor cell lines that possess acquired resistance to anticancer agents. Moreover, we showed that MAO-A expression predicts poor RFS in patients who experienced high-grade ER^−^ or triple negative BC (TNBC) tumors. Collectively our data suggests that a relationship exists between MAO-A and BTIC activity.

## Materials and methods

### Cell culture

Breast tumor cell lines were purchased from the ATCC and propagated as adherent cells or tumorspheres as described previously [8, 9]. The chemically defined media used to culture tumorspheres contains epidermal growth factor (EGF) and fibroblast growth factor 2 (FGF-2).

### Nanostring nCounter

Total RNA was isolated from breast tumor cell lines propagated as adherent cells or as tumorspheres using the Midi Easy RNA isolation kit (Qiagen). Human brain RNA was included as a positive control for MAO-A expression. MAO-A transcript abundance was determined with a custom probe set and normalized by subtracting negative probe counts using Nanostring nSolver software. Normalized expression values are listed in Additional file 1.

### Western blots

Western blots were performed as described previously [9]. To identify MAO-A we used a rabbit monoclonal antibody (Abcam, #ab126751), elicited by a peptide corresponding to amino acids 450–550 of human MAO-A.

### Sphere-forming assays

Clorgyline, tetrindole and pirlindole were purchased from Tocris Bioscience. Sphere-forming assays were carried out as described previously [8, 9].

### Data mining and analysis

Microarray datasets were accessed through the Gene Expression Omnibus (GEO) or Array Express online databases according to the accession codes listed in Table 1. All datasets obtained from the GEO repository were preprocessed as described in their source publications. E-GEOD-28784 dataset was preprocessed by using affy package in R environment with RMA background correction, quantile normalization and median polish summarization methods [13]. Differential expression analysis was performed by using limma package in R [14].

**Table 1:** Transcriptomic analysis of MAO-A expression from mined datasets

Dataset	Comparison	Probe	Fold-change	Adj. p-value
GSE7515	Patient-derived tumorspheres vs primary tumor	212741_at	4.80	5.30E-05
E-MEXP-3982	Docetaxel-resistant MDA-MB-231 vs parental	A_23_P83857	3.34	1.19E-04
E-GEOD-28784	Docetaxel-resistant MDA-MB-231 vs parental	212741_at	1.76	5.40E-03
	Paclitaxel-resistant MDA-MB-231 vs parental	212741_at	2.36	9.07E-04
GSE38376	Lapatinib-resistant SKBR-3 vs parental	ILMN_1663640	2.69	1.02E-14
GSE18912	BMS-536924-resistant MCF-7 vs parental	212741_at	5.46	2.09e-09
GSE19639	LTED MDA-MB-361 vs parental	204388_s_at	4.69	4.10E-12
GSE3542	LTED MCF-7 vs parental	212741_at	3.33	6.30E-10
	Ectopic HER2 expression MCF-7 vs parental	212741_at	5.34	3.00E-11
	Ectopic EGFR expression MCF-7 vs parental	204388_s_at	5.01	3.21E-10
	Ectopic MEK expression MCF-7 vs parental	212741_at	3.62	3.36E-10

Statistical analyses were performed as described in the materials and methods section. The fold change in MAO-A transcript expression between conditions is indicated according to each probe used in the analysis

### Survival analysis

We used the Km Plotter for BC (http://kmplot.com/analysis/) [15]. We selected grade 3 patient tumors that were ER^−^ or of the basal-like intrinsic subtype. Probe [204388_s_at] (MAOA) was used to determine MAO-A expression. For quality control, redundant samples and biased arrays were excluded. Additional file 2 lists the GEO datasets that patient tumors were pooled from.

## Results

### Monoamine oxidase-A expression increases at the transcript and protein level in human breast tumor cells propagated as tumorspheres

To learn whether MAO-A is expressed in human breast tumor cell lines we cultured cell lines representative of all the clinical and molecular subtypes of BC (Additional file 3) in either serum-containing media or media supplemented with defined growth factors [9]. We successfully derived tumorspheres from most of the human breast tumor cell lines that we analyzed. However, consistent with the reports of others [16], the MDA-MB-231 and SKBR-3 cell lines did not form clonal spheres but rather formed cell aggregates and were consequently excluded from our analyses (Additional file 4).

**Fig. 1:**
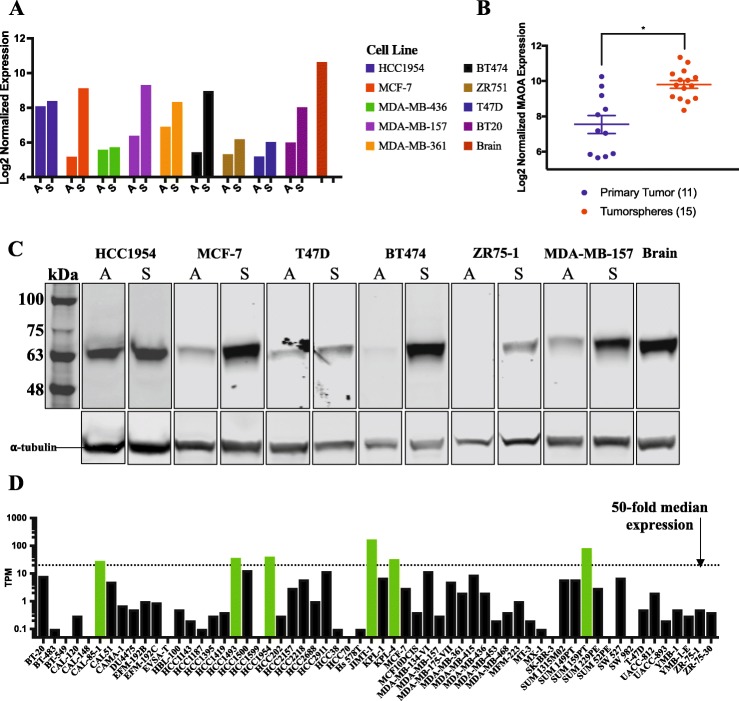
Monoamine oxidase-A expression increases at the transcript and protein level in human breast tumor cells propagated as tumorspheres. **a** Log2 normalized MAO-A transcript counts from Nanostring nCounter analysis of total RNA isolated from human breast tumor cell lines grown adherently (A) or as tumorspheres (S). Human brain RNA was included as a positive control. **b** Log2 normalized MAO-A expression from microarray analysis of primary patient tumor cells propagated as tumorspheres [FC = 4.80; *p* = 5.30E-05]. **c** Western blot analysis of 6 human breast tumor cell lines grown adherently (A) or as tumorspheres (S) with a primary antibody that binds to MAO-A at its approximated molecular weight of 61 kDa. An α-tubulin loading control was included. Lanes were cropped from 3 separate blots as described in Additional file 5. **d** RNA sequencing data from 60 breast tumor cell lines was downloaded from Array Express [E-MTAB-2706]. Transcript abundance (transcripts per million; TPM) was plotted for each cell line. The green bars indicate cell lines that expressed MAO-A at a 50-fold higher level than the median TPM of all samples (black dotted line)

We isolated total RNA from cells propagated under both culture conditions and determined MAO-A transcript abundance using the Nanostring technology. In the majority of breast tumor cell lines MAO-A transcript abundance was higher when cells were propagated as tumorspheres by comparison to those grown as adherent cells (Fig. 1a; Additional file 1).

The availability of transcriptomic data from 11 patient tumor samples and 15 such samples propagated in vitro as tumorspheres allowed us to determine whether the elevated MAO-A expression observed in tumorspheres from established human breast tumor cell lines was reproduced using tumor cells from BC patients [17]. Consistent with previous observations MAO-A expression was significantly higher (fold change [FC] = 4.80; *p* = 5.30E-05) in patient-derived tumor cells propagated as tumorspheres by comparison to the primary breast tumors (Fig. 1b).

To determine whether changes in MAO-A transcript abundance were accompanied by corresponding changes in MAO-A protein expression we prepared protein lysates from 6 breast tumor cell lines cultured as tumorspheres or adherent cells, including at least one cell line from each BC clinical subtype. The abundance of MAO-A protein was higher in lysates isolated from tumorspheres of most of the breast tumor cell lines, except for the HCC1954 cell line, which expressed high levels of MAO-A under both culture conditions (Fig. 1c). Notably the abundance of MAO-A varied among the BC cell lines. These results suggest that increased expression of MAO-A transcripts in breast tumorspheres is accompanied by an increased abundance of the MAO-A protein and that this effect occurs independent of the BC subtype modeled by the cell lines.

To learn what fraction of breast tumor cell lines express MAO-A we examined an RNA-sequencing dataset that includes 60 human breast tumor cell lines that were propagated in serum-containing media [18]. MAO-A was highly expressed in only 6 of these cell lines (Fig. 1d, green bars), which included the ER^−^ EGF receptor 2 overexpressing (HER2^+^) cell lines HCC1954, KPL4 and JIMT1, the TNBC cell lines CAL-85-1 and SUM159PT, and the HCC1493 cell line, which was derived from a male patient (subtype unknown). Hence, in accordance with our observations, MAO-A transcript expression is high in a small fraction of ER^−^ breast tumor cell lines when propagated in serum-containing media.

### Pharmacological inhibition of MAO-A activity reduces the frequency of tumorsphere-forming cells in human breast tumor cell lines

The capacity of cells to form spheres in vitro is a common surrogate assay for BTIC [19]. We and others have shown that agents that reduce BTIC frequency similarly reduce the frequency of tumorsphere-forming cells [20, 21]. Hence, we wondered whether MAO-A activity is required for tumorsphere formation by human breast tumor cell lines. To this end we incubated tumorsphere-derived cells from the MCF-7 and HCC1954 breast tumor cell lines in chemically defined, serum-free media containing serial dilutions of each of 3 different selective MAO-A inhibitors: clorgyline, pirlindole and tetrindole, and 4 days thereafter quantified the number of tumorspheres that arose at each compound concentration. Clorgyline is structurally unrelated to pirlindole or tetrindole, which are structurally related to each other.

**Fig. 2:**
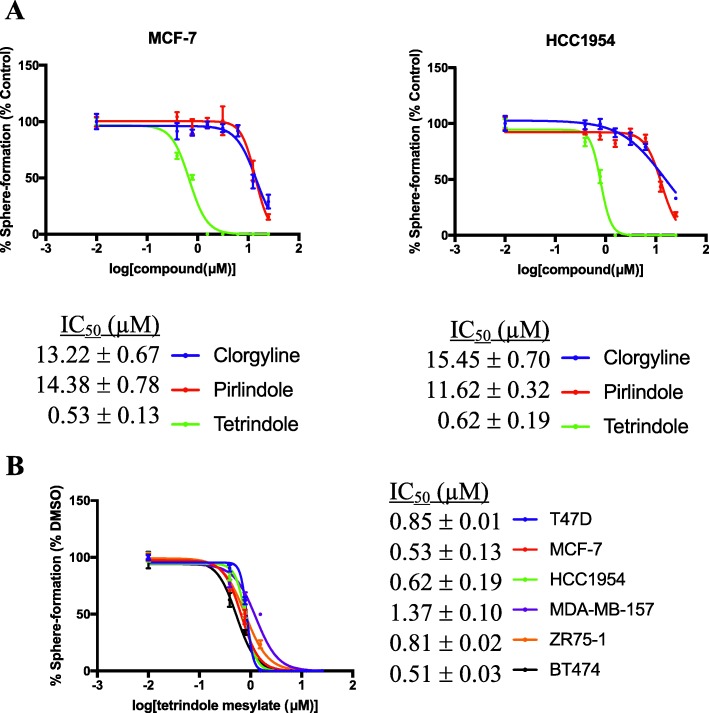
Pharmacological inhibition of MAO-A activity reduces the frequency of tumorsphere-forming cells in human breast tumor cell lines. **a** IC_50_ curves from sphere-forming assays with 3 selective MAO-A inhibitors in the MCF-7 and HCC1954 human breast tumor cell lines. **b** IC_50_ curves for tetrindole using sphere forming assays with a panel of 6 human breast tumor cell lines. Data points represent the number of tumorspheres formed at each concentration, relative to the vehicle treated cells. IC_50_ curves were generated using GraphPad Prism 7.0. Error bars represent standard error from three technical replicates. A value of 0.01 nM was used in IC_50_ calculations as the vehicle-treated control

All 3 compounds reduced the frequency of tumorsphere-forming cells in a dose-dependent fashion by comparison to the vehicle-treated cells, albeit with differing potencies (Fig. 2a). Tetrindole was the most potent inhibitor and hence we expanded its analysis to include all 6 cell lines that we had analyzed by Western immunoblotting, which included at least one cell line from each BC subtype (Fig. 2b). Tetrindole did not appear to have any subtype specificity; its IC_50_ varied between 500 nM and 1500 nM across all the cell lines. These findings suggest that MAO-A activity is required for tumorsphere formation by breast tumor cell lines independent of the BC subtype that they model.

### Increased MAO-A expression is a feature of human breast tumor cell lines resistant to anticancer agents

BTIC are resistant to anticancer agents [2]. Indeed, the frequency of BTIC increases in BC patients after neo-adjuvant chemotherapy due to the sensitivity of the non-tumorigenic tumor cells to cytotoxic agents and the capacity of BTIC to evade these therapies [17]. The increased expression of ATP-binding cassette (ABC) transporters in BTIC may account for their resistance to cytotoxic agents [17, 22]. To learn whether MAO-A expression is correlated with such resistance mechanisms we mined publicly available gene expression profiles of drug-resistant breast tumor cell lines and their drug-sensitive counterparts [23–27] and compared the abundance of MAO-A transcripts (Table 1).

In a study (E-MEXP-3982) of taxane resistance mechanisms in TNBC, a docetaxel-resistant MDA-MB-231 breast tumor cell population was isolated through stepwise exposure to increasing doses of the drug [23]. Acquisition of docetaxel resistance occurred via increased expression and activity of the ABCB1 transporter. Our analysis of the microarray data revealed that MAO-A transcript abundance was higher in docetaxel-resistant MDA-MB-231 cells by comparison to the docetaxel-sensitive parental line (FC = 3.34; *p* = 1.19E-04). We analyzed a dataset from a similar unpublished study (E-GEOD-28784) and found that MAO-A expression is higher in MDA-MB-231 cell populations resistant to docetaxel (FC = 1.76; *p* = 5.40E-03) or paclitaxel (FC = 2.36; *p* = 9.07E-04) by comparison to the parental cell line.

In yet another study (GSE18912) a similar dose-escalation strategy was employed to isolate MCF-7 cells resistant to an insulin growth factor receptor 1 (IGFR1) inhibitor BMS-536924, which resulted from increased expression and activity of the ABCG2 transporter [24]. MAO-A expression was higher (FC = 5.46; *p* = 2.09e-09) in BMS-resistant cells by comparison to the parental MCF-7. Hence multiple studies demonstrate that resistance to common BC therapies, an attribute of BTIC, is associated with increased MAO-A transcript expression.

Whereas ER^+^ breast tumors can be effectively managed with antiestrogen (AE) therapies, long-term estrogen deprivation (LTED) can select for tumor cells that become resistant to these therapies. LTED causes decreased expression of the ER gene cluster and increased expression of receptor tyrosine kinases (RTK) like the epidermal growth factor receptor (EGFR) and human epidermal growth factor receptor 2 (HER2), which provide alternative survival pathways via mitogen activated protein kinases (MAPK) and phosphatidylinositol-3′ kinase (PI3K) [25, 26]. We analyzed the transcriptomic datasets from these two studies and compared the abundance of MAO-A transcripts between LTED breast tumor cells and their parental cell lines.

In the first study (GSE19639), exposure of MDA-MB-361 cells to LTED conditions led to increased PI3K activity, which is part of a phospho-proteomic signature that the authors demonstrated correlates with poor survival of BC patients after neoadjuvant endocrine therapy [25]. We found that MAO-A is up-regulated (FC = 4.69; *p* = 4.10E-12) in LTED MDA-MB-361 cells compared to the parental cell line. A similar study (GSE3542) demonstrated that LTED can be mimicked by ectopic expression of individual components of RTK signaling pathways [26]. Interestingly MAO-A expression was significantly higher in the LTED MCF-7 cells (FC = 3.33; *p* = 6.30E-10) and those MCF-7 cells ectopically overexpressing HER2 (FC = 5.34; *p* = 3.00E-11), MAPK Kinase (MEK) (FC = 3.62; *p* = 3.36E-10), or EGFR (FC = 5.01; *p* = 3.21E-10) compared to controls.

Targeted therapies that inhibit EGFR and HER2 such as lapatinib have been developed and used to treat BC patients with treatment-refractory ER^+^ tumors, but patients often acquire resistance to these agents [27]. We mined the gene expression profiles (GSE38376) of a lapatinib-resistant SKBR-3 breast tumor cell line and found that MAO-A expression was higher in resistant cells (FC = 2.69; *p* = 1.02E-14) by comparison to the parental SKBR-3 cells.

Collectively, these data demonstrate that increased MAO-A expression is associated with several mechanisms of anticancer drug resistance independent of the clinical subtype modeled by the BC cell lines or the anticancer agent being investigated. The findings reported here are novel because we analyzed raw transcriptomic datasets from select studies where MAO-A was not the subject of the investigation.

### MAO-A expression predicts recurrence-free survival in patients who experienced ER^−^ or TNBC tumors

Our analysis of RNA-sequencing data from human breast tumor cell lines revealed that a fraction of TNBC and HER2^+^/ER^−^ breast tumor cell lines express high levels of MAO-A transcripts. Hence, we wondered whether increased MAO-A expression is associated with differential survival of patients who experienced TNBC or ER^−^ tumors. To investigate the latter we used the Km-plotter, which includes the gene expression profiles of thousands of patient primary tumors [15]. We performed two analyses of patients with high-grade tumors by dividing them according to either ER^−^ status or the basal-like (TNBC) subtype.

**Fig. 3:**
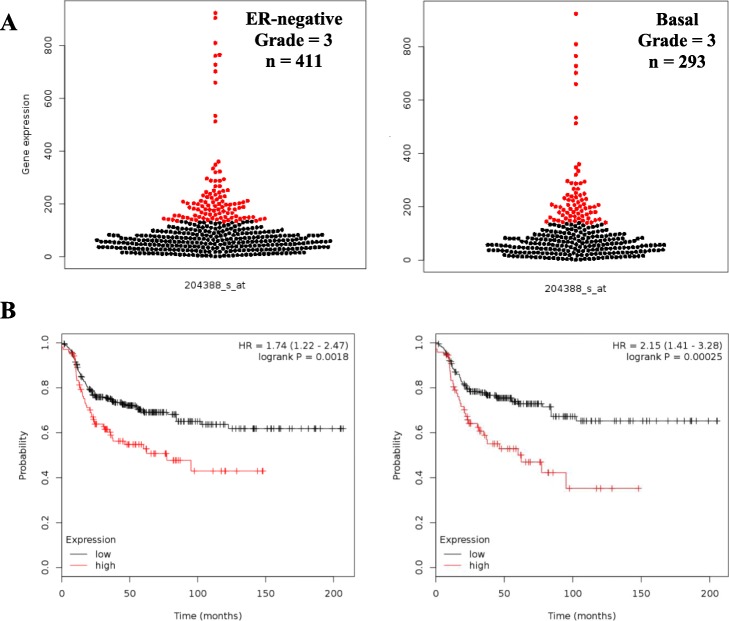
MAO-A expression predicts recurrence-free survival in patients who experienced ER^−^ or TNBC tumors. **a** Beeswarm plot showing MAO-A expression in 411 grade 3 ER^−^ tumors and 293 grade 3 basal-like tumors; red dots show upper quartile separation range. **b** Kaplan-Meier survival curves comparing RFS for high- and low-expressing tumors in ER^**−**^ [HR = 1.74 (1.22–2.47); *p* = 1.8E-03] and basal-like [HR = 2.15 (1.41–3.28); *p* = 2.5E-04] cohorts. Analyses were performed using the Km Plotter for Breast Cancer

Consistent with our observations of breast tumor cell lines and primary breast tumors (Fig. 1), MAO-A expression was low in most of the breast tumors in this analysis (Fig. 3a). Hence to ensure that we were in fact analyzing those tumors with the highest levels of MAO-A transcripts we separated patients based on upper quartile transcript expression levels (Fig. 3a; red dots). In both the ER^−^ and basal-like cohorts elevated MAO-A transcript expression was associated with poor RFS, with hazard ratios of 1.74 (*p* = 1.8E-03) and 2.15 (*p* = 2.5E-04), respectively (Fig. 3b). These results suggest that the fraction of BC patients whose ER^−^ tumors highly express MAO-A are more likely to experience disease recurrence.

## Discussion

Our data suggests that MAO-A expression at the RNA and protein levels is higher in human breast tumor cell lines cultured as tumorspheres by comparison to adherent cells. Inhibition of MAO-A activity with the potent selective inhibitor tetrindole inhibited tumorsphere formation by breast tumor cell lines modeling every BC subtype at similar IC_50_ values thus demonstrating that MAO-A activity plays a functional role in this process. We noted that the level of the MAO-A protein varied widely among the breast cancer cell lines grown in media conducive for tumorsphere formation, yet the IC_50_ of tetrindole was very similar among all the cell lines under these same conditions. This finding implies that MAO-A protein abundance alone may not be a predictor of its activity and that the specific activity of MAO-A might be similar in each of the cell lines. In this regard it is notable that MAO-A activity is regulated by intracellular calcium levels [28], phosphorylation [29] and subcellular localization, all of which may affect its activity [30]. Although the latter may explain the similar potency of tetrindole in different cell lines, further study is warranted to definitively validate tetrindole’s mechanism of action.

Whereas tetrindole is a highly selective MAO-A inhibitor, one study suggested that this compound inhibits calcium ATPase proteins in vitro, albeit with lower potency than the calcium channel blocker verapamil [31]*.* Verapamil was included in the chemical library that we initially screened for compounds that reduce the viability of BTIC-enriched mouse mammary tumor cells [8]. At a concentration of 5 μM, verapamil did not affect tumorsphere formation at all by comparison to the vehicle-treated cells. By contrast, tetrindole inhibited tumorsphere formation with potencies in the high nanomolar range. Hence, whereas we are unable to rule out this alternative hypothesis for the mechanism of action of tetrindole, the inactivity of verapamil in in vitro sphere-forming assays suggests that the latter is unlikely.

We used differential gene expression analyses to show that high MAO-A expression is associated with multiple mechanisms of resistance to several different anticancer agents and is a predictor of poor RFS in patients who experienced ER^−^ or TNBC tumors. Whereas these data were generated using in vitro and in silico analyses, they provide a compelling rationale for examining the efficacy of selective MAO-A inhibitors in preclinical models of breast cancer.

A recent shRNA screen was performed using tumorspheres isolated from the TNBC cell line, SUM149 [32]. BTIC-enriched SUM149 cells were transduced with a pooled lentivirus shRNA library including multiple shRNAs targeting MAO-A and then propagated as tumorspheres. Thereafter next-generation DNA sequencing of shRNA barcodes revealed that those shRNAs targeting MAO-A were statistically significantly depleted during the culturing of the tumorspheres. This finding provides independent functional evidence in agreement with our data demonstrating that MAO-A plays a required role in tumorsphere formation and that reducing MAO-A transcript abundance or activity with selective inhibitors is sufficient to inhibit this process.

RNA-sequencing data from 60 human breast tumor cell lines grown in serum-containing media revealed that 6 cell lines express very high levels of MAO-A transcripts by comparison to all other cell lines. JIMT1, HCC1954 and KPL4 were derived from ER^−^/HER2^+^ tumors of patients that were refractory to HER2-targeted therapy [33, 34] and these cell lines are resistant to RTK inhibitors such as lapatinib [35]. Interestingly, whereas SKBR-3 are sensitive to lapatinib [35], they display elevated expression of MAO-A after acquiring resistance to this agent (Table 1). Consistent with the latter findings, increased MAO-A protein expression in clinical specimens predicts poor overall survival in patients who experienced HER2^+^ BC [36].

We established that increased MAO-A transcript expression is associated with ABC-transporter-mediated resistance to taxane chemotherapeutics and predicts poor prognosis in patients who experienced high-grade ER^−^ or TNBC tumors. Several studies have proposed that increased ABC transporter expression and activity endows BTIC with resistance to cytotoxic anticancer therapies [22]. Indeed, residual breast tumors after neoadjuvant chemotherapy comprise an increased frequency of BTIC [17] and overexpress several ABC transporters by comparison to surrounding non-tumor tissue [37].

The expression of BTIC markers in breast tumors is also associated with poor clinical outcomes [3]. For example, metastatic breast tumors and those with increased histological grade have a higher frequency of CD44^+^/CD24^−/low^ and ALDH^+^ BTIC. Hence the poor survival associated with high MAO-A expression in primary tumors might be related to an increased frequency of therapy-resistant BTIC in those tumors.

We found that MAO-A is differentially upregulated in breast tumor cells that have acquired ER-independence via LTED or ectopic expression of RTK. Studies have established that estrogen-independent growth of breast tumor cells increases the frequency of BTIC and that of tumorsphere-forming cells [38, 39]. Notably, the chemically defined media used to culture tumorspheres lacks estrogen and contains the RTK-stimulating growth factors EGF and FGF-2 [8, 9]. We suspect that culturing ER^+^ breast tumor cell lines as tumorspheres mimics the conditions required for ER-independent growth. Indeed propagating MCF-7 cells as tumorspheres induces a microRNA-orchestrated silencing of the ER and a complete epithelial-to-mesenchymal transition resulting in the stable enrichment of CD44^Hi^/CD24^Lo^ BTIC [40]. Moreover, MCF-7 tumorsphere-derived cells comprise a higher fraction of BTIC compared to adherently-grown cells and express a gene signature that includes MAO-A and predicts poor response to AE therapy [6].

Analogous findings have been observed in prostate tumor cells where long-term androgen deprivation leads to increased MAO-A expression and activity [11]. Reactive oxygen species produced by MAO-A enzymatic activity facilitate hormone-refractory neuroendocrine differentiation, which reportedly increases TIC activity [12]. Interestingly, the first evidence that MAO-A contributes to BC progression demonstrated that the increasing degree of malignancy in chemically-induced rat breast tumors is associated with elevated MAO-A enzymatic activity [10, 41]. High-grade adenocarcinomas displayed increased serotonin-specific enzymatic activity by comparison to benign hyperplasia, as established by Lineweaver-Burk analysis of MAO-A kinetics. Hence, a role for MAO-A in TIC activity and BC progression is consistent with the observations of others.

## Conclusion

We have established that MAO-A activity is required for tumorsphere formation by human breast tumor cell lines. Our sphere-forming assays have identified tetrindole as a potential novel anticancer agent. We also found that increased expression of MAO-A is a feature of breast tumor cell lines that have acquired anticancer drug resistance and the tumors of patients that experienced poor RFS, implying that MAO-A expression might be of prognostic value in BC. It is particularly intriguing that altered MAO-A expression occurred in cell lines modeling every BC clinical subtype given the substantial molecular heterogeneity that exists among the subtypes. Collectively, our observations suggest that further study of the connection between MAO-A and BTIC activity is warranted. The establishment of MAO-A as a marker of therapy resistance and disease recurrence in high-grade breast tumors and as a potential target for treatment would have broad implications in breast cancer research.

## Supplementary information

**Additional file 1.** Normalized MAO-A mRNA counts from Nanostring nCounter analysis. MAO-A abundance was determined from total RNA using Nanostring nCoutner and custom probe sets. mRNA read counts were normalized by subtracting negative probe counts using Nanostring nSolver software. Human Brain RNA was included as a positive control.

**Additional file 2.** GEO datasets used for RFS survival analysis.

**Additional file 3.** Human breast tumor cell lines used in this study. The clinical and molecular subtype of each cell line is indicated.

**Additional file 4.** MCF-7 human breast tumor cells form bona fide tumorspheres, whereas MDA-MB-231 form cellular aggregates. Images taken of MDA-MB-231 cells (top) and MCF-7 cells (bottom) grown in chemically defined media as tumorspheres. Tumorspheres were imaged at 100X magnification and the scale bar represents 100 μm. The arrows demarcate examples of a bona fide tumorspheres (solid arrows) and cellular aggregates (dashed arrows). (B) Examples of each structure shown at a higher magnification (200X).

**Additional file 5 **Western blots used to create Fig. 1c. We cropped lanes from each blot to create Fig. 1c. MAO-A and α-tubulin bands for HCC1954 A and S lanes were taken from **Blot 1**, imaged at a low exposure **(A)**. MAO-A bands from MCF-7 A and S, MDA-MB-157 A and S, and mouse brain were taken from the **Blot 2,** taken at a low exposure **(B)**. MAO-A bands from T47D A and S and ZR75–1 A and S were also taken from **Blot 2**, imaged at a higher exposure **(C).** α-tubulin bands from MCF-7 A and S, MDA-MB-157 A and S, T47D A and S, ZR75–1 A and S, and mouse brain were all taken from **Blot 2**, imaged at a low exposure **(D)**. MAO-A and α-tubulin bands from BT474 A and S were taken from **Blot 3**, imaged at a low exposure **(E)**.

## Data Availability

All publicly available datasets are available through the Gene Expression Omnibus or Array Express according to the accession codes that are listed in Table 1. The associated studies are cited in the results section where applicable. Normalized MAO-A mRNA expression counts from the Nanostring analysis are reported in Additional file 1.

## Abbreviations

ABC: ATP-binding cassette
AE: Anti-estrogen
BC: Breast cancer
BTIC: Breast tumor-initiating cell
EGF/R: Epidermal growth factor / receptor
ER: Oestrogen receptor
FGF2: Fibroblast growth factor 2
HER2: Human epidermal growth factor receptor 2
LTED: Long-term oestrogen deprivation
MAO-A: Monoamine oxidase-A
MAPK: Mitogen-activated protein kinase
MEK: MAPK kinase
PI3K: Phosphatidylinositol-3′ kinase
RFS: Recurrence-free survival
RTK: Receptor tyrosine kinase
SERT: The serotonin transporter
TIC: Tumor initiating cell
TNBC: Triple-negative BC
