# Supplementary material for: Monoamine oxidase-A activity is required for clonal tumorsphere formation by human breast tumor cells
Source: Cell Mol Biol Lett. 2019 Nov 12;24:59. doi: 10.1186/s11658-019-0183-8 (PMC6852929; doi:10.1186/s11658-019-0183-8)
Supplement: Supplementary file 1 — Additional file 1. Normalized MAO-A mRNA counts from Nanostring nCounter analysis. MAO-A abundance was determined from total RNA using Nanostring nCoutner and custom probe sets. mRNA read counts were normalized by subtracting negative probe counts using Nanostring nSolver software. Human Brain RNA was included as a positive control. [file 11658_2019_183_MOESM1_ESM.pdf]

Additional File 1. Normalized MAO-A mRNA counts from Nanostring nCounter analysis.

**Cell Lines**	**Adherent**	**Tumorspheres**
BT20	63.53	258.63
BT474	42.68	496.09
MCF-7	35.97	550.82
MDA-MB-361	118.65	320.39
MDA-MB-436	47.54	52.69
T47D	36.32	64.63
ZR75-1	39.66	71.93
MDA-MB-157	82.81	627.16
HCC1954	270.2	330.55
Brain	1566.64	

MAO-A abundance was determined from total RNA using Nanostring nCoutner and custom probe sets. mRNA read counts were normalized by subtracting negative probe counts using Nanostring nSolver software. Human Brain RNA was included as a positive control.
