# Supplementary material for: Monoamine oxidase-A activity is required for clonal tumorsphere formation by human breast tumor cells
Source: Cell Mol Biol Lett. 2019 Nov 12;24:59. doi: 10.1186/s11658-019-0183-8 (PMC6852929; doi:10.1186/s11658-019-0183-8)
Supplement: Supplementary file 2 — Additional file 2. GEO datasets used for RFS survival analysis. [file 11658_2019_183_MOESM2_ESM.pdf]

Additional File 2. GEO datasets for RFS survival analysis.

Cohort	Datasets
ER^- (n = 411)	GSE11121, GSE1456, GSE16446, GSE16716, GSE17907, GSE19615 GSE20271, GSE20711, GSE21653, GSE2990, GSE31519, GSE3494, GSE37946, GSE42568, GSE45255, GSE4611, GSE7390, GSE9195
Basal (n = 293)	GSE11121, GSE1456, GSE16446, GSE16716, GSE19615, GSE20711, GSE21653, GSE2990, GSE31519, GSE3494, GSE37946, GSE42568, GSE45255, GSE4611, GSE7390, GSE9195
