# Supplementary material for: Monoamine oxidase-A activity is required for clonal tumorsphere formation by human breast tumor cells
Source: Cell Mol Biol Lett. 2019 Nov 12;24:59. doi: 10.1186/s11658-019-0183-8 (PMC6852929; doi:10.1186/s11658-019-0183-8)
Supplement: Supplementary file 3 — Additional file 3. Human breast tumor cell lines used in this study. The clinical and molecular subtype of each cell line is indicated. [file 11658_2019_183_MOESM3_ESM.pdf]

Additional File 3. Human breast tumor cell lines used in this study.

Cell Line	Molecular Subtype	ER	PR	HER2
MCF-7	Luminal	+	+	-
BT474	Luminal	+	+	+
T47D	Luminal	+	+	-
ZR75-1	Luminal	+	+	-
MDA-MB-361	Luminal	+	+	+
HCC1954	Basal A	-	-	+
MDA-MB-157	Basal B	-	-	-
MDA-MB-436	Basal B	-	-	-
BT20	Basal A	-	-	-

The clinical and molecular subtype of each cell line is indicated.
