# Supplementary figures and images for: Monoamine oxidase-A activity is required for clonal tumorsphere formation by human breast tumor cells
Source: Cell Mol Biol Lett. 2019 Nov 12;24:59. doi: 10.1186/s11658-019-0183-8 (PMC6852929; doi:10.1186/s11658-019-0183-8)

**A MDA-MB-231**

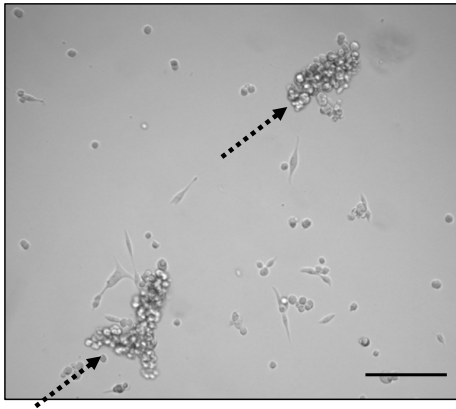

**B**

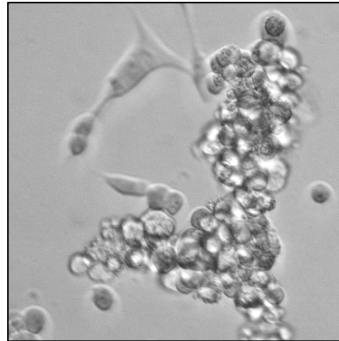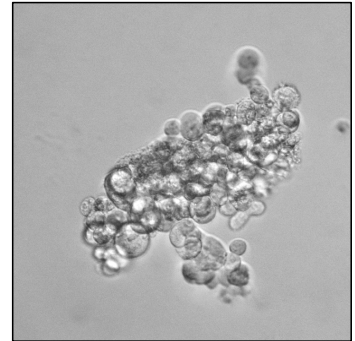

**MCF-7**

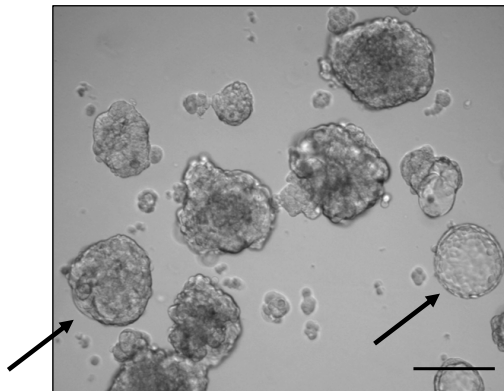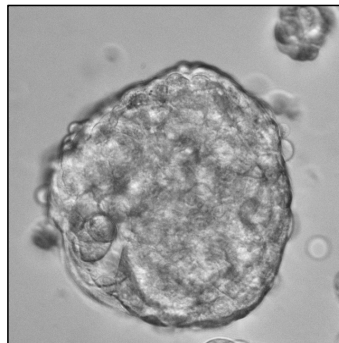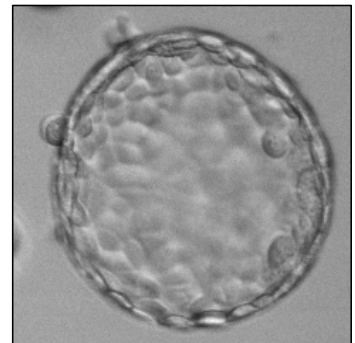

Supplement: Supplementary file 4 — Additional file 4. MCF-7 human breast tumor cells form bona fide tumorspheres, whereas MDA-MB-231 form cellular aggregates. Images taken of MDA-MB-231 cells (top) and MCF-7 cells (bottom) grown in chemically defined media as tumorspheres. Tumorspheres were imaged at 100X magnification and the scale bar represents 100 μm. The arrows demarcate examples of a bona fide tumorspheres (solid arrows) and cellular aggregates (dashed arrows). (B) Examples of each structure shown at a higher magnification (200X). [file 11658_2019_183_MOESM4_ESM.pdf]
