# Supplementary material for: Monoamine oxidase-A activity is required for clonal tumorsphere formation by human breast tumor cells
Source: Cell Mol Biol Lett. 2019 Nov 12;24:59. doi: 10.1186/s11658-019-0183-8 (PMC6852929; doi:10.1186/s11658-019-0183-8)
Supplement: Supplementary file 5 — Additional file 5 Western blots used to create Fig. 1c. We cropped lanes from each blot to create Fig. 1c. MAO-A and α-tubulin bands for HCC1954 A and S lanes were taken from Blot 1, imaged at a low exposure (A). MAO-A bands from MCF-7 A and S, MDA-MB-157 A and S, and mouse brain were taken from the Blot 2, taken at a low exposure (B). MAO-A bands from T47D A and S and ZR75–1 A and S were also taken from Blot 2, imaged at a higher exposure (C). α-tubulin bands from MCF-7 A and S, MDA-MB-157 A and S, T47D A and S, ZR75–1 A and S, and mouse brain were all taken from Blot 2, imaged at a low exposure (D). MAO-A and α-tubulin bands from BT474 A and S were taken from Blot 3, imaged at a low exposure (E). [file 11658_2019_183_MOESM5_ESM.pdf]

A

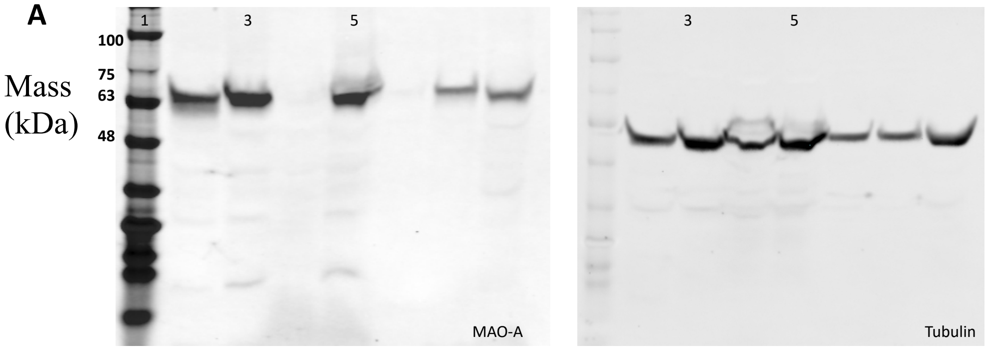

Blot 1 Low Exposure
Lanes cropped into Figure
1 - Marker
3 - HCC1954 Spheres
5 - HCC1954 Adherent

3 - HCC1954 Spheres
5 - HCC1954 Adherent

B

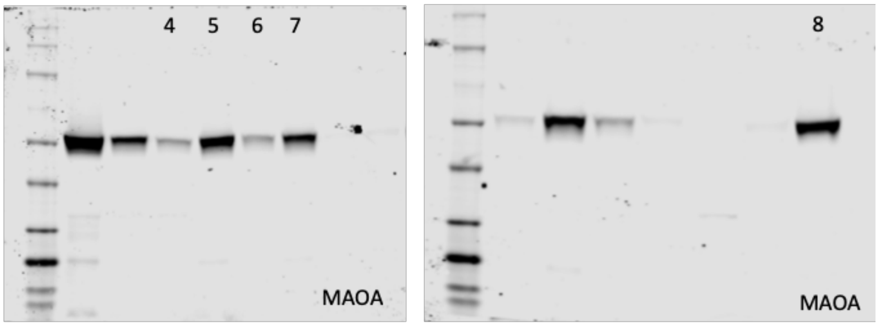

Blot 2 Low Exposure
Lanes cropped into Figure
4 – MCF-7 Adherent
5 – MCF-7 Spheres
6 – MDA-MB-157 Adherent
7 – MDA-MB-157 Spheres

8 – Mouse Brain

C

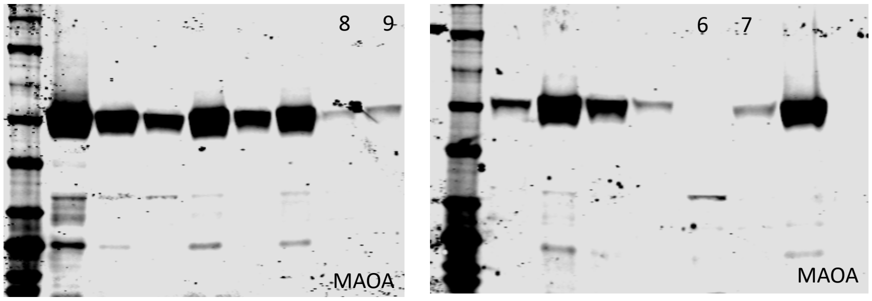

Blot 2 High Exposure
Lanes cropped into Figure
8 - T47D Adherent
9 - T47D Spheres

6 - ZR75-1 Adherent
7 - ZR75-1 Spheres

D

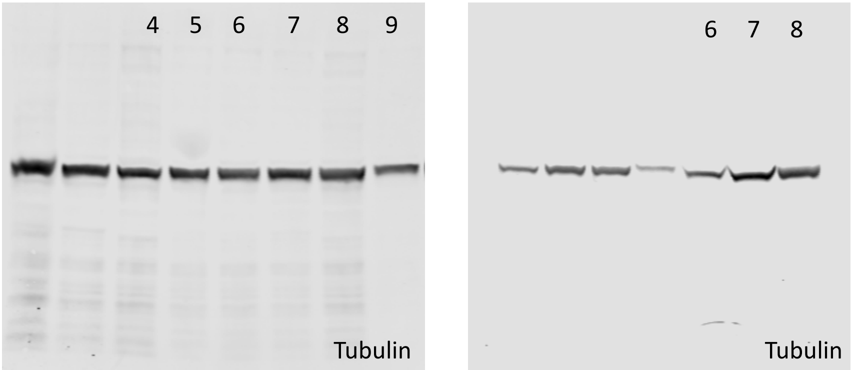

Blot 2 Low Exposure
Lanes cropped into Figure
4 – MCF-7 Adherent
5 – MCF-7 Spheres
6 – MDA-MB-157 Adherent
7 – MDA-MB-157 Spheres
8 - T47D Adherent
9 - T47D Spheres

6 - ZR75-1 Adherent
7 - ZR75-1 Spheres
8 - Mouse Brain

E

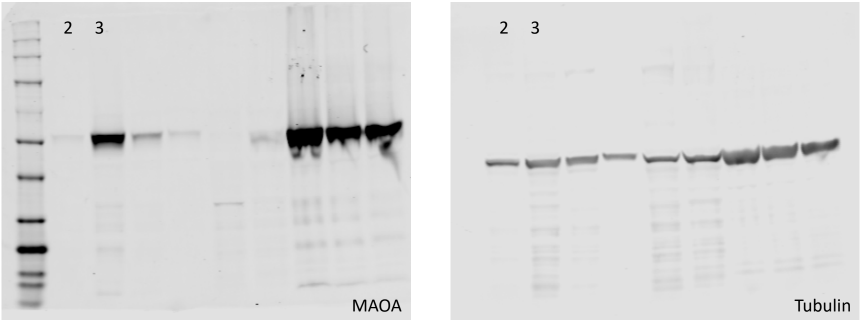

Blot 3 Low Exposure
Lanes cropped into Figure
2 - BT474 Adherent
3 - BT474 Spheres

2 - BT474 Adherent
3 - BT474 Spheres
